# State-Dependent Modulation of Slow Wave Motifs towards Awakening

**DOI:** 10.3389/fncel.2017.00108

**Published:** 2017-04-24

**Authors:** Daisuke Shimaoka, Chenchen Song, Thomas Knöpfel

**Affiliations:** ^1^Neuroinformatics Japan Center (DS), RIKEN Brain Science InstituteSaitama, Japan; ^2^Institute of Ophthalmology, University College LondonLondon, UK; ^3^Laboratory for Neuronal Circuit Dynamics, Imperial College LondonLondon, UK; ^4^Centre for Neurotechnology, Institute of Biomedical Engineering, Imperial College LondonLondon, UK

**Keywords:** cortical circuit dynamics, slow cortical waves, global wave events, spatiotemporal motifs, genetically encoded voltage indicators, optical voltage imaging

## Abstract

Slow cortical waves that propagate across the cerebral cortex forming large-scale spatiotemporal propagation patterns are a hallmark of non-REM sleep and anesthesia, but also occur during resting wakefulness. To investigate how the spatial temporal properties of slow waves change with the depth of anesthetic, we optically imaged population voltage transients generated by mouse layer 2/3 pyramidal neurons across one or two cortical hemispheres dorsally with a genetically encoded voltage indicator (GEVI). From deep barbiturate anesthesia to light barbiturate sedation, depolarizing wave events recruiting at least 50% of the imaged cortical area consistently appeared as a conserved repertoire of distinct wave motifs. Toward awakening, the incidence of individual motifs changed systematically (the motif propagating from visual to motor areas increased while that from somatosensory to visual areas decreased) and both local and global cortical dynamics accelerated. These findings highlight that functional endogenous interactions between distant cortical areas are not only constrained by anatomical connectivity, but can also be modulated by the brain state.

## Introduction

Slow-wave sleep (SWS, also referred to as non-REM sleep) is characterized by large-amplitude slow waves in the electroencephalogram (EEG; Blake, 1937). These slow cortical waves are prominent during anesthesia too (Steriade et al., 1993b,c; Mohajerani et al., 2010) and similar cortical activities were also described in humans (Sachdev et al., 2015) and in mice during the resting wakefulness state in various cortical areas (somatosensory, Poulet and Petersen, 2008; Gentet et al., 2010; visual, Polack et al., 2013; and auditory cortex, Schneider et al., 2014).

Cortical slow waves reflect the alternating transition between periods of neuronal hyperpolarization (silence state of cortical neuronal firing) and depolarization (active state of cortical neuronal firing; Steriade et al., 1993a, b,c, 2001; Cowan and Wilson, 1994; Contreras and Steriade, 1995; Timofeev et al., 2001; Chauvette et al., 2010). The alternation between these two distinct states of the cortical network, each lasting hundreds of milliseconds, results in a peak in the EEG and local field potential (LFP) power spectra in the range of 0.1–5 Hz. Intracellular recordings from anesthetized and awake rodents demonstrated that, when the LFP displays slow waves, neighboring cortical pyramidal neurons alternate between active and silent states synchronously (Crochet and Petersen, 2006; Mahon et al., 2006; Poulet and Petersen, 2008; Gentet et al., 2010; Okun et al., 2010). This synchronization can recruit cortical areas of variable sizes, generating local or global slow waves that can propagate spatially across cortical regions (Massimini et al., 2004) Slow waves may be locally restricted, with neighboring areas being engaged in a different pattern of active/inactive state transition or in a longer lasting “desynchronized” state (Poulet and Petersen, 2008; Gentet et al., 2010). Previous work suggests that more localized and more global waves form a continuum rather than a categorical dichotomy (Nir et al., 2011). Slow waves propagate with preferred directions along major anatomical pathways (Massimini et al., 2004; Murphy et al., 2009; Botella-Soler et al., 2012) and individual waves may be driven by distinct cortical origins (Massimini et al., 2004; Volgushev et al., 2006; Riedner et al., 2007; Kurth et al., 2010; Nir et al., 2011).

However, little is known about the change in spatiotemporal properties of slow waves at different levels of anesthesia (or the brain states). One such property that could potentially be brain state-dependent is the spatiotemporal features of these waves, including their propagation pattern and speed. Also of particular interest is whether common spatial motifs are present throughout different brain states. We hypothesize that the spatial propagation pattern of the waves remains intact along major anatomical pathways irrespective of the brain state, while the temporal properties could change according to the local cortical state, based on the findings that the slow wave propagates along the cortex polysynaptically (Steriade et al., 1993b,c).

To address these hypotheses, we used VSFP Butterfly 1.2, a genetically encoded voltage indicator (GEVI), to optically image voltage transients generated by mouse layer 2/3 pyramidal neurons across one or both cortical hemispheres during recovery from barbiturate anesthesia. The advantage of this recovery from anesthesia model is the well-known variation of “brain states” as seen in the cortical EEG and as exploited in previous studies (e.g., Constantinople and Bruno, 2011; Ecker et al., 2014; Scott et al., 2014). We focus on the depolarizing half waves that recruited at least 50% of the imaged cortical area. We characterized the spatiotemporal features of these global wave events (GWEs) and investigated how these features may change from deep anesthesia towards the waking state. Based on a data-driven clustering approach, GWE patterns were classified into several distinct motifs that were largely conserved across brain states from deep anesthesia to awakening. With the emergence of the awakening, the incidence of individual motifs changed systematically. Moreover, we observed an acceleration of slow wave dynamics within each local cortical area, as well as its propagation speed across distant cortical areas. These spatiotemporal properties provide key characteristics towards a mechanistic understanding of how slow wave activity can potentially contribute to their implied roles in functions such as learning and memory (Marshall et al., 2006; Rasch et al., 2007) and homeostasis of network activity (Cirelli et al., 2005; Tononi, 2009).

## Materials and Methods

### Animal Preparation

The gene encoding VSFP Butterfly 1.2 was introduced into mouse layer 2/3 excitatory neurons either by *in utero* electroporation (ICR; Akemann et al., 2012, 2013) or via transgenic approaches (Camk2a-tTA:RasGrf2-dCre:Ai78, Madisen et al., 2015). The transgenic mice Ai78 is a VSFP-B reporter line that is doubly regulated by Cre and tTA. For the detail of the intersectional transgenic approach used, see Madisen et al. (2015). To target neurons in cortical layer 2/3 *in utero* electroporation was performed at embryonic day 15.5, and transgenic mice Ai78 was crossed with a RasGrf2-dCre driver line. Due to scattering and absorption of the blue excitation light (Al-Juboori et al., 2013), the dominant fraction of the fluorescence signal was recorded from layer 2/3. Experimental procedures for the *in utero* electroporated mice were approved by the Institutional Animal Care and Use Committee of the RIKEN Wako Research Center (Japan) and were conducted according to the US National Institutes of Health guidelines for animal research. Experimental procedures for the transgenic mice were performed in accordance with the UK Animal Scientific Procedures Act (1986) under UK Home Office Personal and Project licenses following ethical review. Mice (aged 2–6 months, either sex) were implanted with a head post and a thinned skull cranial window over the dorsal part of one hemisphere (mouse no. 1–3; VSFP expressed via electroporation) or both hemispheres (mouse no. 4–6; VSFP expressed via transgenic approach) as described previously (Akemann et al., 2012, 2013). In brief, the animal was deeply anesthetized with pentobarbital sodium (40–90 mg/kg i.p.), a head post was implanted onto the thinned mouse skull and secured using self-cure adhesive resin cement (Super-Bond C&B, Sun Medical, Japan). The thinned skull was reinforced by a cover glass using a cyanoacrylate adhesive (Drew et al., 2010) or a layer of adhesive resin cement topped by a thin layer of clear nail polish (Sofroniew et al., 2015). Animals were singly-housed post-surgery under normal light/dark cycle.

### Voltage Imaging

For imaging sessions, after at least 7 days of recovery from the cranial window implant surgery, mice were re-anesthetized with pentobarbital sodium (40 mg/kg i.p.) and head-fixed via the implanted head post in a custom-made holding frame, with body temperature maintained by means of a feedback-controlled heat pad (Fine Science Tools, USA). Pentobarbital was used for its ability to induce cortical slow wave (Steriade et al., 1993b), and its slow recovery time (typically 1–2 h) to enable the monitoring of slow wave across different anesthesia depths. Following *i.p*. injection of pentobarbital sodium, the animals proceeded through different levels of anesthesia, which was assessed by independent measurement of EEG activity, chest wall movement and heart rate. Since these measures matched well, heart rate was used as a proxy measure of anesthesia depth in subsequent imaging experiments (Janssen et al., 2004). The level of anesthesia was also confirmed by the animal’s responsiveness to touch and observable spontaneous whisker and limb movements between imaging sessions. The imaging session was performed for 121.8 ± 7.9 min after induction of anesthesia (mean and SE across six mice) until the animal started to show spontaneous movements.

Image acquisition was performed with a dual emission wide-field epifluorescence macroscope equipped with two synchronized CCD cameras (Sensicam, PCO, Germany), using high power halogen lamps (Moritex, BrainVision, Japan) and macroscope lenses (Planapo 1.0× M-series Leica, Germany). Excitation light was passed through a FF01–483/32–25 BP optical filter and 515LP beam splitter (Semrock). Emission light was split by a 580LP dichroic mirror, FF01–542/27–25 optical filter for mCitrine fluorescence, and BLP01–594R-25 optical filter for mKate2 fluorescence. Image sequences of 60–240 s duration with approximately 60 s interval were acquired at 50 frames/s at 320 × 240 pixel resolution. An imaged pixel corresponded to 33 × 33 μm of a projected cortical area. In each mouse, the ROI was defined by the area that exhibited clear reporter fluorescence (exceeding green autofluorescence as observed in wild type mice by a factor of ~5). The electroporated mice (mouse no. 1–3) expressed the VSFP in one hemisphere whereas the transgenic mice (no. 4–6) expressed the voltage reporter in both hemispheres. The ROI sizes were 18 mm^2^ (mouse no. 1–3, one hemisphere) and 41 mm^2^ (mouse no. 4–6, both hemispheres). ROIs they were registered to the location of the bregma, facilitating comparisons across animals.

### VSFP Signal Separation

Imaging data were analyzed using MATLAB (R2014b) with the Image and Signal Processing Toolboxes (Mathworks Inc, USA). The voltage imaging signal was calculated as the ratio of mKate2 (FRET acceptor) to mCitrine (FRET donor) fluorescence, taken after equalization of heartbeat-related fluorescence modulation (Akemann et al., 2012). Notably this equalization procedure has been to shown to effectively discount vascular artifacts such as heart-beat oscillation and slow (<1 Hz) fluctuation due to hemodynamics in anesthetized (Akemann et al., 2012) and awake (Carandini et al., 2015) conditions. The resulting ratiometric sequences of voltage maps were then smoothed spatially (2D gaussian, *σ* = 49.5 μm) and temporally (Chebyshev Type II band-path filtering 0.5–9 Hz, function *imfilter*, MATLAB, Mathworks Inc, USA). We excluded the first 10 s of each sequence to remove any effects of sensory stimulation (e.g., shutter noise and excitation light onset) related to the initiation of imaging sequence acquisition.

### Wave Detection

Individual slow waves were detected similar to the previously described methods for LFP and intracellular recordings of slow waves (Mölle et al., 2002; Nir et al., 2011). Waves were detected as positive deflections between two zero crossings for each pixel within the imaged cortical area and slow waves in particular are defined whereby these positive deflections are separated by 0.06–1.0 s. When slow waves were detected in more than 10% of all pixels and reached 50% of all pixels within the ROI (at any time point during the half wave event), we defined this time epoch as GWE. Events initially detected as single GWEs but containing multiple well-isolated peaks in the fraction of recruited pixels were separated into individual GWEs at the time of trough between peaks. The trough was detected as the time of local minimum when slow wave was detected in less than 50% of all pixels within the ROI. Note that our detection scheme allows GWEs to propagate as some pixels may be recruited at the wave front while others may have terminated their half waves during the GWE. Hence, GWEs are dynamic in size and localization.

In a small subset of data (especially when animals start to show spontaneous movements), the ratiometric signal showed strong deviations from baseline (~5 times larger dR/R than slow wave activity). We assigned these deflections as movement artifacts as they were also prominent in the summated donor and acceptor fluorescence (D + A; a voltage-independent report of the dynamics in hemeoglobin concentration and oxygenation in the optical path through the tissue that is more sensitive to correlated movement-induced fluorescence signals than the anticorrelated ratiometric voltage signal). We therefore excluded time epochs when D + A exceeded 2 SD. This process rejected 4.5 ± 0.4 (SD) % of total frames.

### Propagation Pattern of Global Wave Events

We then analyzed the propagation patterns of detected GWEs, similar to a previously described method for human EEG analysis (Ito et al., 2007). For each pixel and frame, the voltage time course was converted into instantaneous phase (via Hilbert transform, function *hilbert*, MATLAB, Mathworks Inc, USA). The instantaneous phase was then converted into phase difference relative to the instantaneous phase averaged across ROI (we confirmed that the result is not affected by the choice of reference pixels). Finally, the sequences of relative phase maps of each GWE were time-averaged, yielding a single spatial phase pattern map (SPPM) for each GWE.

### Classification of Global Wave Events

To see if slow waves have distinctive propagation patterns, similarity between SPPMs was calculated as the Euclidean distance of the phase of the complex voltage signals (amplitude and phase) across all pixels. For each animal, we computed the distances between all the SPPMs observed from deep anesthesia till awakening. Having observed signs of distinctive patterns in SPPMs hierarchical cluster trees, SPPMs were classified into three clusters using k-means clustering (Hutt et al., 2003). We observed that separation of SPPMs into 2–5 clusters gave similar results (Supplementary Figure S2).

### Measurement of Propagating Speed

To calculate propagation speeds, voltage maps were additionally spatially filtered (2D Gaussian, *σ* = 132 μm). For each pixel recruited during a GWE, we computed the time of peak amplitude. For each map of peak time, we then computed the local spatial gradient for each pixel, and determined the propagation speed as the median of these gradients.

### Depth of Anesthesia

To see any differences in slow wave properties influenced by anesthesia depth, heartbeat frequency was used as a proxy of anesthesia depth. In each animal, data epochs when heart rate frequency was the lowest (typically 6 Hz at the beginning of the recording session) was assigned as deeply anesthetized state, whereas higher frequencies (typically 10–12 Hz occurring during the last part of the session and consistent with the heartbeat frequency of awake behaving mice under the scope) were assigned as awakening state. The awakening was also confirmed by responsiveness to touch and observable spontaneous whisker and limb movements between recordings.

### Statistical Analysis

To investigate the difference in slow wave properties between different levels of anesthetized states, we used non-parametric two-sided tests (Wilcoxon rank-sum test, Chi-square test for independence, Wilcoxon signed-rank test) that make no assumption about the underlying data distributions. We did not predetermine sample sizes by power analysis, but our sample sizes are comparable to those reported in previous wide-field voltage imaging studies using mice (Mohajerani et al., 2013; Scott et al., 2014).

## Results

### Voltage Imaging of Cortical Activities and Detection of Slow Waves

**Figure 1:**
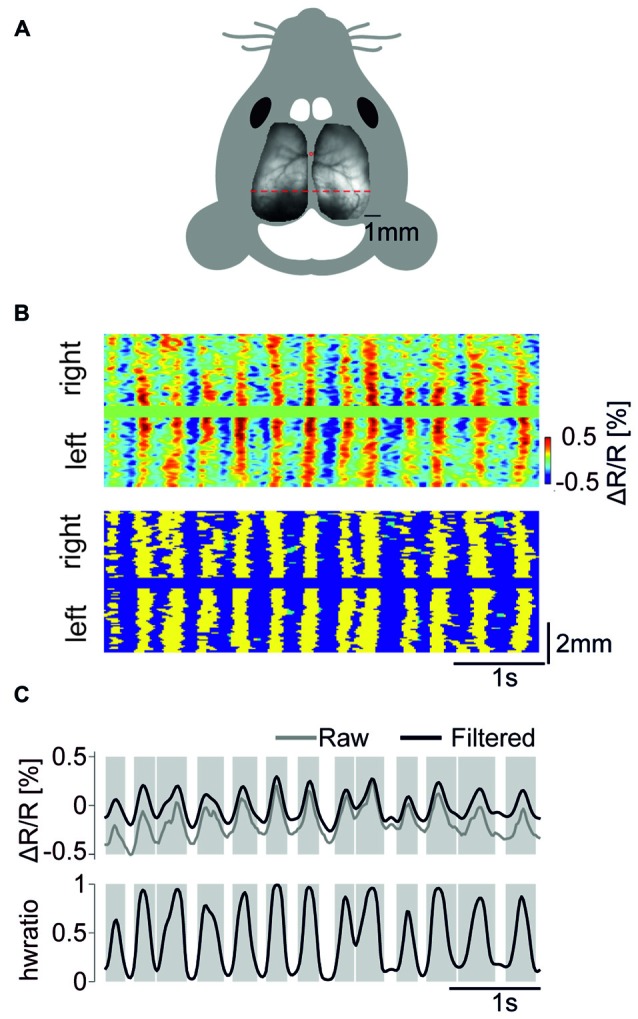
**Detection of global wave events (GWEs). (A)** Fluorescence image of a transgenic mouse brain (mCitrine channel) mounted on schematic outline of the mouse head. **(B)** Upper panel. Scan of color coded, high-pass (>0.5 Hz) filtered voltage signals along the red dotted line in **(A)**. Lower panel. Detected GWEs marked as yellow. Regions marked as green indicates depolarizing half wave events that were not classified as engaging in a GWE. **(C)** Upper panel. Voltage trace (raw signal in gray, high pass (>0.5 Hz) filtered in black), averaged across both hemispheres. Lower panel. Fraction of pixels of both hemispheres exhibiting depolarizing half waves. Gray shaded epochs indicate times of individual GWEs. Example data are from animal #6.

We performed transcranial optical voltage imaging using the GEVI VSFP Butterfly 1.2 targeted to layer 2/3 pyramidal cells either via *in utero* electroporation (Akemann et al., 2010, 2012), imaging over one hemisphere, group 1 (mouse no. 1–3); or using gene targeted mice based on the Cre-lox system (Madisen et al., 2015), imaging over both hemispheres, group 2 (mouse no. 4–6). Our wide-field epifluorescence imaging approach captured the membrane voltage averaged over tissue volumes that are projected onto each pixel, allowing neural activity to be recorded across the cortex with high spatiotemporal resolution (<100 μm, spatially oversampled at 33 × 33 μm^2^; limited by light scattering, 20 ms, Figure 1A). We imaged cortical activity of these mice throughout the recovery from pentobarbital anesthesia (40 mg/kg i.p.). We first detected depolarizing half waves pixel-by-pixel using a zero crossing algorithm, and we then detected periods during which >50% of the pixels within the imaged cortical area are simultaneously engaged in depolarizing half waves and termed these events as GWEs (Figure 1B). Figure 1C (Upper) shows an example time course trace of the voltage signal (averaged across both hemispheres; mouse no. 6) and the fraction of engaged pixels. Mean frequency of GWEs across animals was 2.35 ± 0.12 Hz.

### Global Wave Events can be Classified into Distinct Motifs

**Figure 2:**
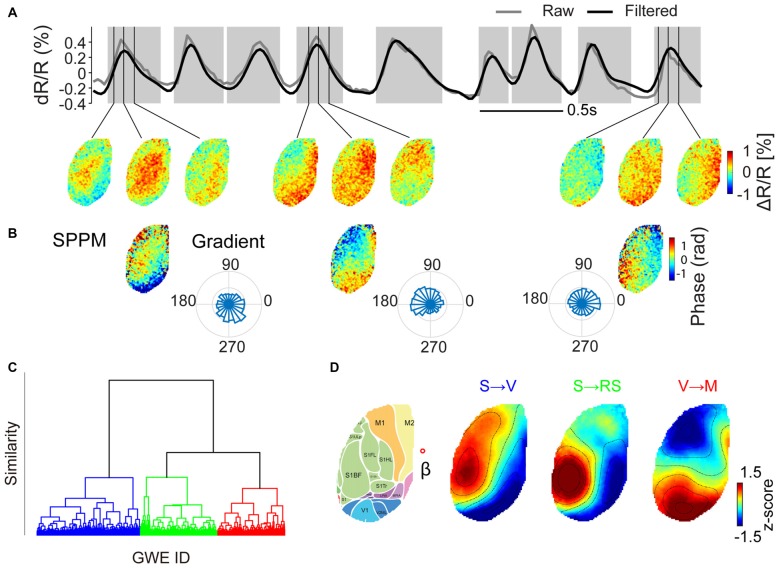
**Clustering of GWEs into distinct motifs. (A)** Upper panel. The time course of voltage signal averaged across both hemispheres (raw signal in gray, high pass (>0.5 Hz) filtered signal in black). Epochs shaded in gray indicate times of individual GWEs. Lower panel. Voltage maps at times as indicated by vertical lines. **(B)** Spatial phase pattern maps (SPPMs), representing propagation direction of each event and probability distributions of directions of wave events, computed as mean local spatial gradients during GWE for each pixel. **(C)** Hierarchical cluster tree, obtained by hierarchical clustering applied to SPPMs to the whole dataset of animal #3 from deep anesthesia through awakening, recorded in one session (trees for the other five animals are shown in Supplementary Figure S1). The vertical axis indicates distance (similarity) between nodes. **(D)** SPPM cluster centroids for the case when all GWEs are classified into one of three motifs (“S → V”, “S → RS” and “V → M”), as indicated in different colors in **(C)**. Right image shows the approximate location of cortical regions (Kirkcaldie, 2012), overlaid on the Citrine fluorescence image. Red open circle indicates the approximate location of Bregma (β) (Scale bars = 1 mm).

We first examined quantitatively whether GWEs can be classified into stereotyped patterns. Figure 2A shows an example of voltage maps obtained at three time points for three example GWEs. We noted that for each of the three selected GWEs the spatiotemporal pattern is clearly dissimilar, originating from different sites and propagating in different directions. We then explored if there are repeated patterns from a finite set of motifs. To address this we computed the similarity between SPPMs (Figure 2B) from deep anesthesia through awakening, and generated hierarchical dendrograms for each animal. In the six animals investigated (three of group 1 and three of group 2), the structure of the resulting dendrograms suggested that individual GWEs can be classified into one of several stereotypical spatiotemporal patterns (Figures 2C,D, and Supplementary Figure S1). Indeed, when we classified all detected events into three groups using k-means clustering, the centroids of the three clusters show distinct spatial pattern and propagation direction (Figure 2D). Partition using more than four clusters would sometime generate “private” motifs that were not common to all animals (Supplementary Figure S2). We found comparable cluster centroids across all six animals analyzed, and categorized them according to their propagation patterns into three motifs. Motif “S→V” shows propagation from somatosensory cortex and primary motor cortex to visual cortex; motif “S → RS” shows propagation from somatosensory areas to visual and retrosplenial cortex; and motif “V → M” shows propagation from visual cortex and secondary motor cortex to primary motor cortex.

### Global Wave Event Spatial Patterns, but Not their Prevalence, Are Conserved Over Different Anesthesia Depths

**Figure 3:**
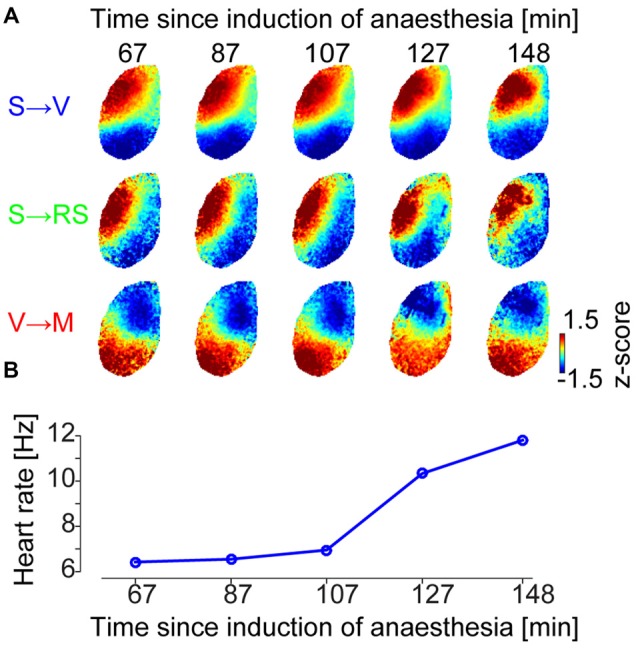
**Conserved repertoire of distinct wave motifs at different levels of anesthesia. (A)** Centroids of SPPM clusters at different times between drug delivery and wakening (Example data from animal #2, centroids for the other five animals are shown in Supplementary Figure S3). Each centroid is normalized by its mean and standard deviation across pixels for visualization purpose. **(B)** Heartbeat frequency, as a proxy for wakefulness of the animal, represented as temporally aligned with **(A)**.

We next investigated whether these GWE spatial phase pattern motifs (cluster patterns) are conserved across different levels of anesthetized states. To address this, we examined whether the spatial phase pattern of each motif changes during the transition from deep to light anesthesia (Figure 3A and Supplementary Figure S3). We found that the spatial phase pattern of individual motifs is largely conserved through different anesthetized states. Indeed, we found high spatial correlation between centroids obtained at different states during recovery from anesthesia, computed within the same motif (0.85 ± 0.05 (SE of median) across animals and motifs). This correlation was significantly higher than correlations computed between centroids of different motifs across anesthesia depths (*n* = 18 motifs across animals, *p* = 8.4 × 10^−7^, Wilcoxon rank-sum test), and correlations between centroids of different motifs within anesthesia depth (*n* = 18 motifs across animals, *p* = 1.7 × 10^−5^, Wilcoxon rank-sum test).

**Figure 4:**
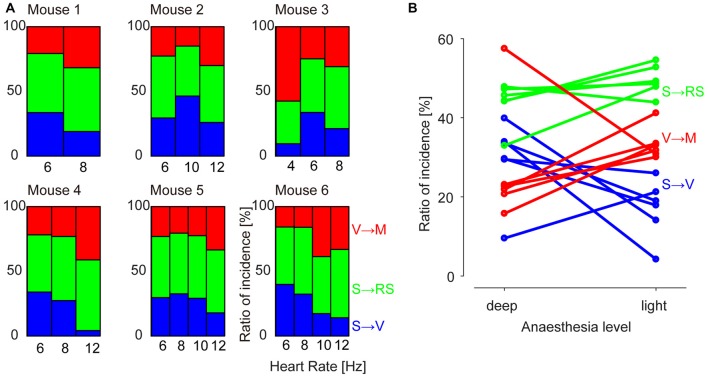
**Prevalence of motifs at different levels of anesthesia. (A)** Percentage of incidence of occurrences of each motif (“S → V”, “S → RS” and “V → M”). Values are normalized to the total number of GWEs for each recording epoch. **(B)** Summary plot across animals and motifs. Each sample point represents incidence of a motif of an animal.

We then calculated the prevalence of the GWE spatial phase motifs as a function of time through different anesthesia levels. In contrast to their spatial patterns, the prevalence of each GWE motif systematically evolved over time with the decline of anesthesia level (Figure 4; *n* = 4945, 4730, 3371, 2414, 1355, 2618 GWEs, chi-square independence test, *p* < 2.4 × 10^−4^ in all six experiments). Notably, prevalence of the “V → M” motif increased in five out six animals accompanying their recovery from anesthesia (Residual analysis, *n* = 1114, 1130, 1476, 583, 373, 519 “V → M” GWEs, *p* < 2.3 × 10^−5^ in each of five experiments), while prevalence of the “S → V” motif significantly decreased in four out of six animals (*n* = 1544, 1367, 525, 735, 334, 887 “S → V” GWEs, *p* < 6.7 × 10^−7^ in each of four experiments). “S → RS” motif remained relatively constant, as only two out six animals showed significant increase (*n* = 2287, 2233, 1370, 1096, 648, 1212 “S → RS” GWEs, *p* < 2.4 × 10^−4^ in each of two experiments).

### Propagation Speed of GWEs Increases with Decline of Anesthesia Depth

**Figure 5:**
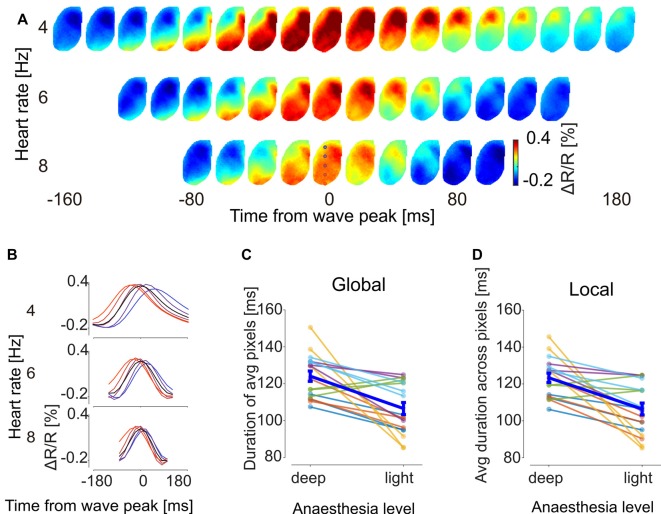
**GWE dynamics accelerate towards recovery from anesthesia. (A)** Sequences of voltage maps at different levels of anesthesia. For each motif individual GWEs were aligned to their voltage peak time and averaged. Shown is motif “V → M” of animal #3 (full dataset shown in Supplementay Figure S4). Colored circles at 3rd row at 0 ms indicates location of pixels used to show time traces in **(B)**. **(B)** Time traces of the aligned GWE shown in **(A)**. Black traces and colored traces show spatial averages and individual pixel responses, respectively. **(C)** Global duration of the GWE, computed from time trace of average across pixels. Each sample point represents duration of a motif of an animal. **(D)** Local duration of the GWE, computed for each time trace of each pixel and then averaged across pixels.

**Figure 6:**
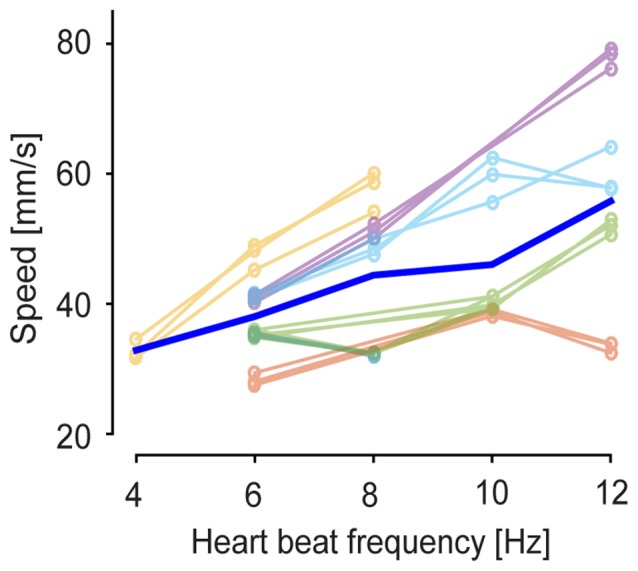
**Wave propagation speed at different anesthetized states.** Propagation speed on cortical surface was estimated at the single-event level (see “Materials and Methods” Section). Thin lines represent mean propagation speed of GWEs for each anesthetized state. Blue thick line indicates average across six experiments and three motifs.

The clustered SPPMs were calculated as temporal averages over GWE duration, leaving open the possibility that their temporal features may change with brain state. To investigate this possibility, voltage map time course was averaged across the wave events for each of the three SPPM motifs for each 60 s recording sequence by aligning GWEs to the peak of their spatially averaged voltage signal. The resulting GWE motif voltage map sequences indicated that the propagation direction and spatial patterns of GWE motifs are conserved across different levels of anesthesia (Figure 5A) confirming the SPPM-based analysis (Figure 3A). On the other hand, the wave front appears to travel faster with decreased anesthesia depth (Figure 5A), indicating an acceleration of circuit dynamics. To quantitatively examine this observation, we computed the durations of the spatially averaged voltage transients for each motif and found that duration decreases as the animal transits towards awakening (Figure 5B black trace, duration was measured as full width at half maximum). This was confirmed by population analysis (Figure 5C; *n* = 18, from six animals and three motifs, *p* = 8.7 × 10^−4^, Wilcoxon signed-rank test). Faster dynamics of the spatially averaged voltage transient may be caused by an accelerated local circuit dynamics and/or a faster activity propagation speed (and hence reduced dispersion). To investigate these possibilities, we computed the durations of the motif’s voltage transients for each pixel (Figure 5B, colored traces) and then averaged over the local durations. This analysis revealed that the duration of the voltage transients decreases in each cortical area, indicating that slow wave time course also accelerated locally (Figure 5D; *n* = 18, from six animals and three motifs, *p* = 6.2 × 10^−4^, Wilcoxon signed-rank test). We then calculated the propagation speed for each GWE motif at different anesthesia level, and found that the speed of slow-wave propagation increases during recovery of anesthesia (Figure 6; *n* = 18, from six animals and three motifs, *p* = 5.4 × 10^−4^, Wilcoxon signed-rank test). Together, our findings show that both local and global cortical dynamics accelerated towards awakening.

## Discussion

We developed an analysis strategy to identify and quantitatively characterize slow wave events transcranially imaged using a GEVI targeted to layer 2/3 cortical pyramidal neurons. Slow waves can occur locally, especially at awake state (Vyazovskiy et al., 2011), but here we focused on slow waves that involve (and traverse) large parts of the cortical surface, termed GWEs. The core findings of our analysis are: (i) the presence of distinct spatiotemporal motifs of GWEs with three principal motifs dominating throughout different anesthetic brain states; (ii) the systematic change of principal motif prevalence during recovery from anesthesia; and (iii) the temporal compression of the motifs both at the local circuit level as well as at the cortex-wide level towards awakening.

### Distinct Spatiotemporal Motifs of Slow Waves

We generated hierarchical dendrograms of spatial phase pattern similarities and found that they indicate the presence of distinct GWE motifs. This discreteness of GWE propagation pattern is in line with previous slow wave studies in humans (Massimini et al., 2004) and in mice (Mohajerani et al., 2013). Inspection of the dendrograms prompted us to separate the GWEs into three categories based on their propagation patterns. Intracranial recordings in human previously revealed a bimodal distribution of depolarizing wave durations, but while this seems to point towards a classification into just two groups, we did not see such grouping in our data set (Botella-Soler et al., 2012). The hierarchical dendrogram suggests that the GWEs can also be expanded into a larger set of motifs (Supplementary Figure S2), but would start to yield “private” motifs that are not common to all animals.

Our observations of distinct motifs overlap with a recent study by Mohajerani et al. (2013) who found motifs of connectivity patterns calculated from correlated spontaneous activity between seed pixels and remote cortical areas, where the motifs recapitulated activation patterns triggered by prior sensory stimulation. Mohajerani et al. (2013) found marked resemblance between the spatial pattern of the motifs and anatomical connectivity (Oh et al., 2014), implying that the cortico-cortical network guides the propagation pattern of the motif. This view is in line with our findings, where the motifs’ spatial structure is conserved across anesthesia depth (Figures 3,  5). However, the functional interactions between distant areas are not simply hard-wired and only bound to the anatomical connectivity, but undergo significant modulation by the brain state: incidence of the motifs (Figure 4) and their propagation speed (Figure 6) changes as the animal wakes up.

We did not relate our motifs to sensory responses but, interestingly, origins of the identified sources of slow wave arise from sensory regions: somatosensory (“S → V” and “S → RS”, anteroposterior direction), and visual (“V → M”, posteroanterior direction) cortices (Figure 2D). Our imaging window did not extend to the very lateral and frontal regions such as temporal and frontal association cortices, thus it is possible that additional motifs originating beyond our dorsal window also exist that may contribute to higher cognitive integration. Unlike previous studies in mice (Ruiz-Mejias et al., 2011; Sheroziya and Timofeev, 2014) and human (Massimini et al., 2004) where traveling waves frequently originate in the frontal cortices and propagate in the anteroposterior direction, we observed the opposite propagation direction more commonly in the present study, but this is not entirely contradictory and could be dependent on anesthetic level. In fact, anteroposterior waves (motif “S → V”) are also more prevalent during deep anesthesia, and at decreasing anesthesia level, the reduced prevalence of anteroposterior waves is accompanied by a consistent increase in the posteroanterior waves (motif “V → M”), and relatively constant “S → RS” motif (Figure 4). Our study shows for the first time that GWE spatiotemporal motifs were robustly found over different levels of anesthesia and only their relative prevalence evolved during the course of approaching wakefulness. By investigating the change in slow wave dynamics throughout the course of decreasing anesthesia depths, our findings again demonstrate that the slow wave propagation dynamics is not always fixed, but is under the influence of brain state. The experiments reported here do not provide evidence for the physiological mechanism underlying the brain-state dependency of motif prevalence.

### Accelerated Circuit Dynamics Towards Awakening

While the same GWE spatial phase pattern motifs were robustly found over different levels of anesthesia, the dynamics of the GWE clearly accelerated during recovery of anesthesia and reached their maximum with awakening. As animals woke up, local slow wave operated faster (Figure 5D) and the GWE propagated faster as well (Figure 6). A potential explanation for the acceleration of local dynamics would be an increased excitatory and inhibitory synaptic conductance, which in turn decreases the effective integration time of the postsynaptic cell, an effect known as synaptic shunting (Bernander et al., 1991; Häusser and Clark, 1997). In addition, state dependent neuromodulation may act on intrinsic excitability and synaptic integration. The slow wave propagation speed (<0.1 mm/ms) is much slower than axonal conduction velocities of cortical neurons (>1 mm/ms), suggesting polysynaptic mechanism, as also indicated by experiments using local pharmacological inactivation (Golomb and Amitai, 1997). Given a polysynaptic mechanism, the increased propagation speed may simply be a consequence of faster local circuit dynamics. Indeed, activity dependent modulation of inter-regional axonal conduction velocities has been described (Swadlow et al., 1980; Chida et al., 2015). However, this is unlikely to be the only mechanism underlying our observation, since it does not affect local circuit dynamics. Indeed, differential modulation of synaptic conductance at awake state has been reported in visual cortex (Haider et al., 2013). One of the proposed mechanisms generating alternating cycles of depolarization and hyperpolarization during slow wave activity is by the interplay between recurrent excitation and a negative feedback, due to slow activity-dependent potassium currents (Golomb and Amitai, 1997; Sanchez-Vives and McCormick, 2000; Compte et al., 2003). In this scheme, increasing excitatory and inhibitory synaptic conductance accelerates global propagation speed, and decreases duration of the depolarized state. Anesthesia (and barbiturate-mediated anesthesia in particular) likely affects GABAergic mechanisms (conductance and driving force). Intuitively speculated, increased inhibition could accelerate circuit dynamics (e.g., via shunting inhibition, see above) or decrease the frequency of oscillatory events if the depolarizing phase is terminated by an accumulative build-up of inhibition. Alterations of inhibitory mechanism between subclasses of inhibitory neurons (e.g., disinhibition via depressed activity of one subclass) may produce similarly difficult-to-predict net effects on slow wave frequency and propagation.

### Slow Waves and their Reflectance of Functional Connectivity

Our study supports the notion that the spatiotemporal dynamics of slow waves reflect the functional connectivity between intra-cortical circuits (Mohajerani et al., 2013) as well as the dynamic state (Scott et al., 2014).

Finally, the present work also supports the notion that circuit activities that emerge as slow waves use specific information pathways that are normally used for sensorimotor integration in the awake state. This feature is consistent with the presumed role of slow waves in memory consolidation, synaptic homeostasis and the restorative functions of sleep (Cirelli et al., 2005; Marshall et al., 2006; Rasch et al., 2007; Tononi, 2009; Walker, 2009; Diekelmann and Born, 2010). In addition, our findings highlight that the dynamic functional interactions between distant cortical regions are not only dependent on connectivity as defined by functional anatomical mapping, but such cortico-cortical communication is also subject to brain state modulation.

## Author Contributions

CS generated new experimental data. DS and TK analyzed data. TK, DS and CS wrote manuscript.

## Funding

This work has been supported by intramural funds from RIKEN and Imperial College, and grants from the HFSPO and NIH. DS is supported by Simons Foundation (325512).

## Supplementary Material

The Supplementary Material for this article can be found online at: http://journal.frontiersin.org/article/10.3389/fncel.2017.00108/full#supplementary-material

## Conflict of Interest Statement

The authors declare that the research was conducted in the absence of any commercial or financial relationships that could be construed as a potential conflict of interest.
