# Supplementary material for: State-Dependent Modulation of Slow Wave Motifs towards Awakening
Source: Front Cell Neurosci. 2017 Apr 24;11:108. doi: 10.3389/fncel.2017.00108 (PMC5401891; doi:10.3389/fncel.2017.00108)
Supplement: Supplementary file 1 [file Presentation_1.pdf]

Supplementary Material

Accelerated Dynamics of Slow Wave Motifs During Awakening

Daisuke Shimaoka[1, 2], Chenchen Song[3] and Thomas Knöpfel [2, 3, 4]

*** Correspondence:** Thomas Knöpfel: tknopfel@knopfel-lab.net

1 Supplementary Figures

Figure S1

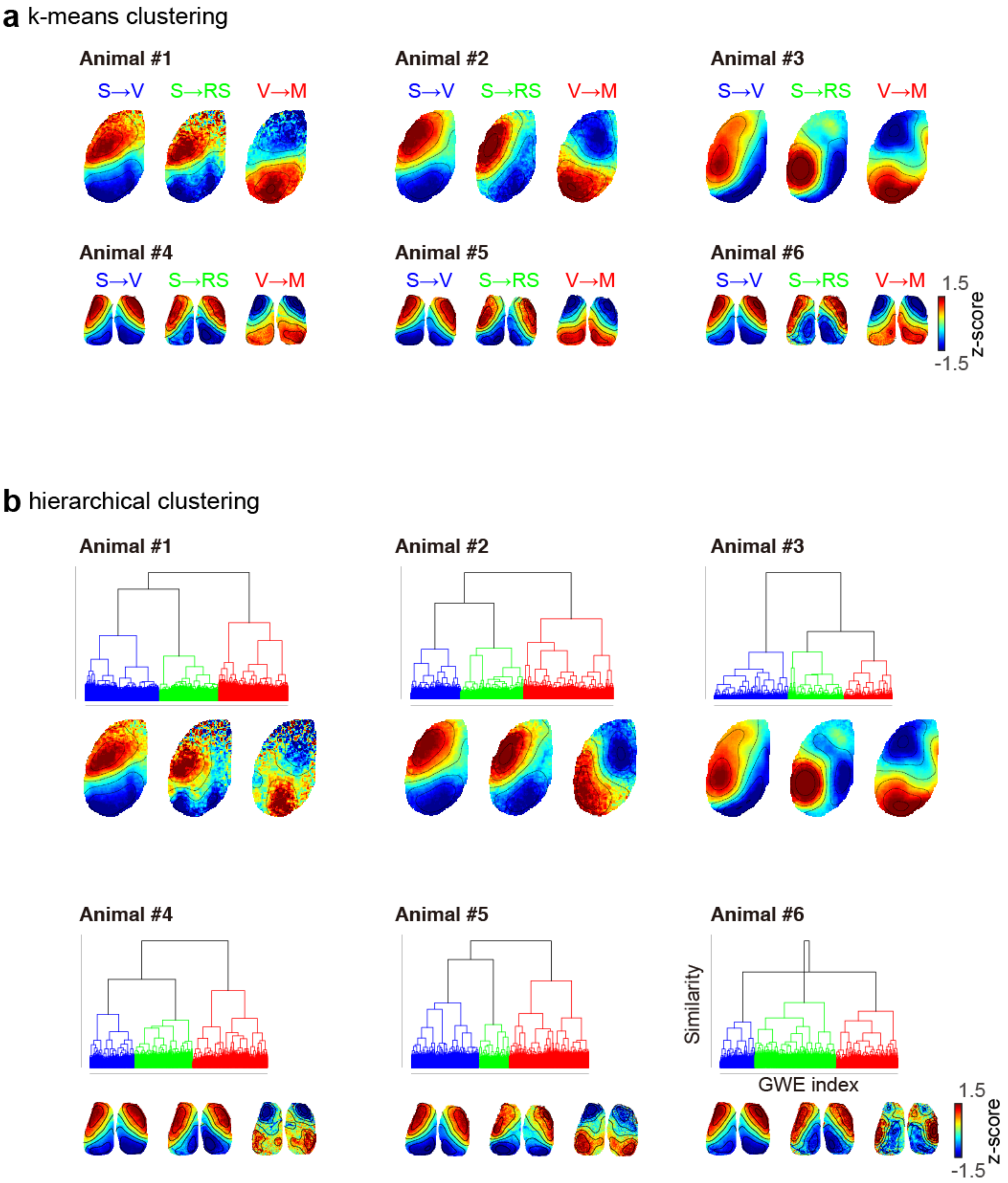

Figure S1, related to Figure 2. Classification of GWEs via **a** k-means clustering and **b** utilizing hierarchical dendrogram.

Figure S2

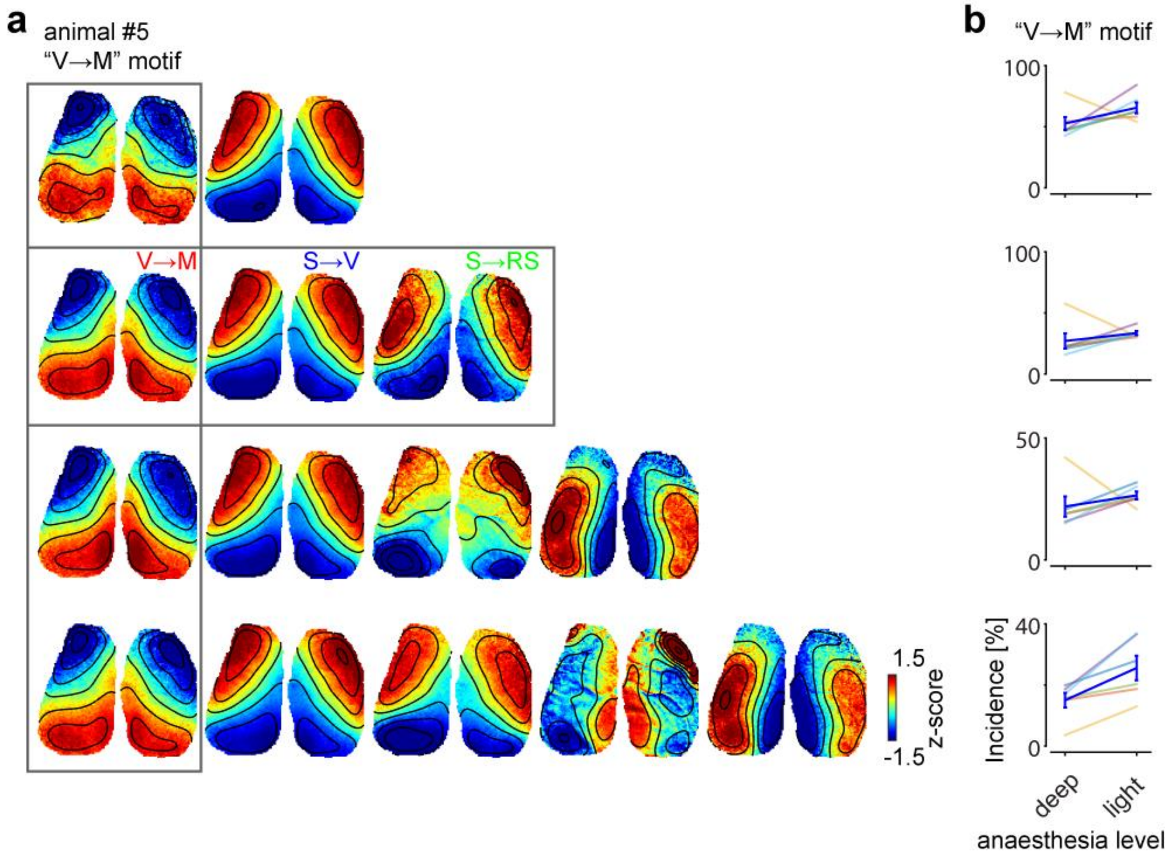

Figure S2, related to Figure 2. Result when GWEs are classified into different number of clusters. **a** Cluster centroids with different cluster numbers. In each row the left most SPPM corresponds to motif ("S→V", "S→RS" and "V→M"). **b** Prevalence of motif "V→M" at the beginning and end of imaging session. Thin lines represent results from each experiment. Blue thick line indicates average and S.E. across 6 experiments.

Figure S3

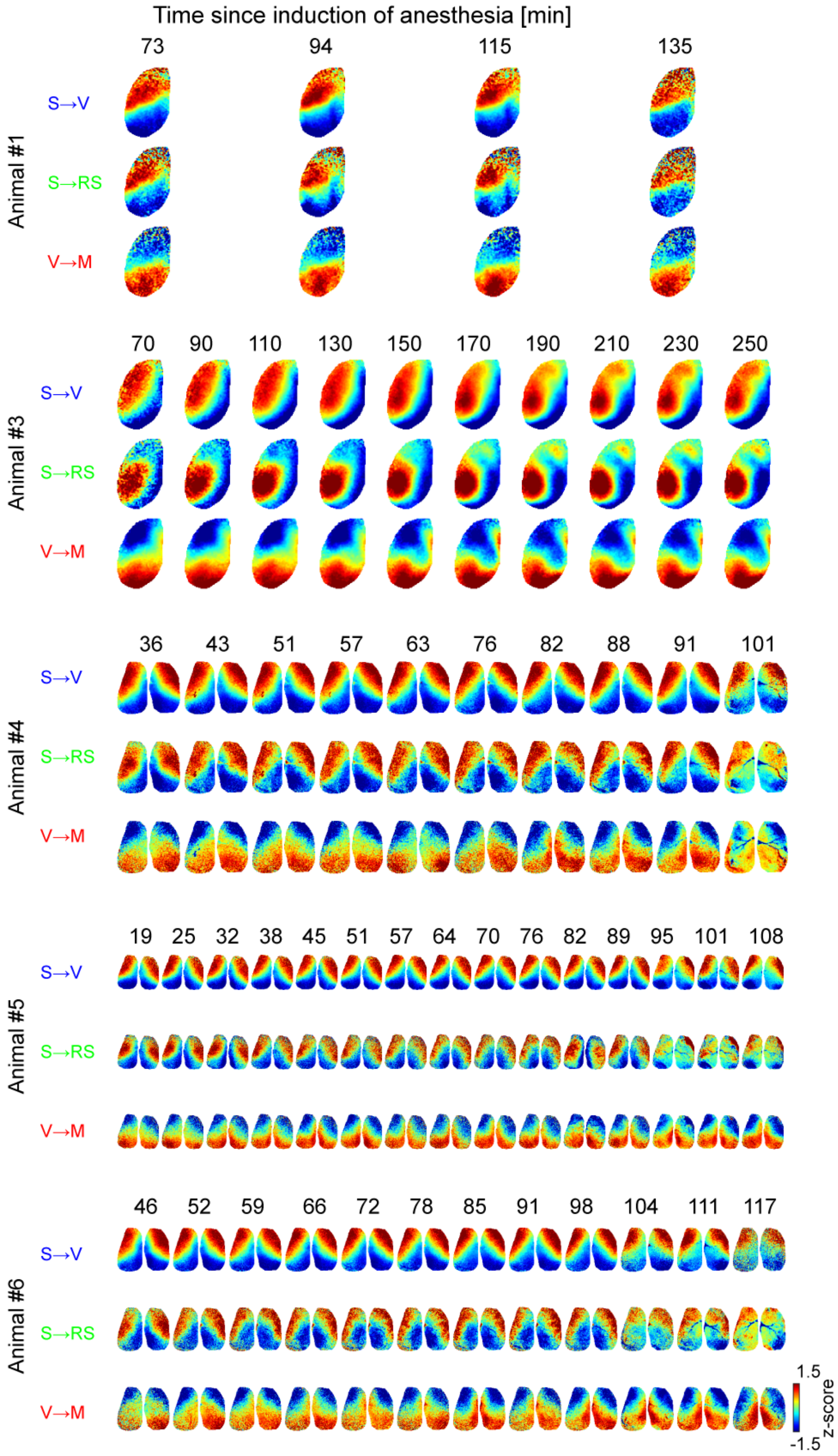

Figure S3, related to Figure 3. Expansion of Figure 3a for all animals. Centroids of SPPM clusters at different times between drug delivery and wakening. Each centroid is normalized by its mean and standard deviation for visualization purpose.

Figure S4

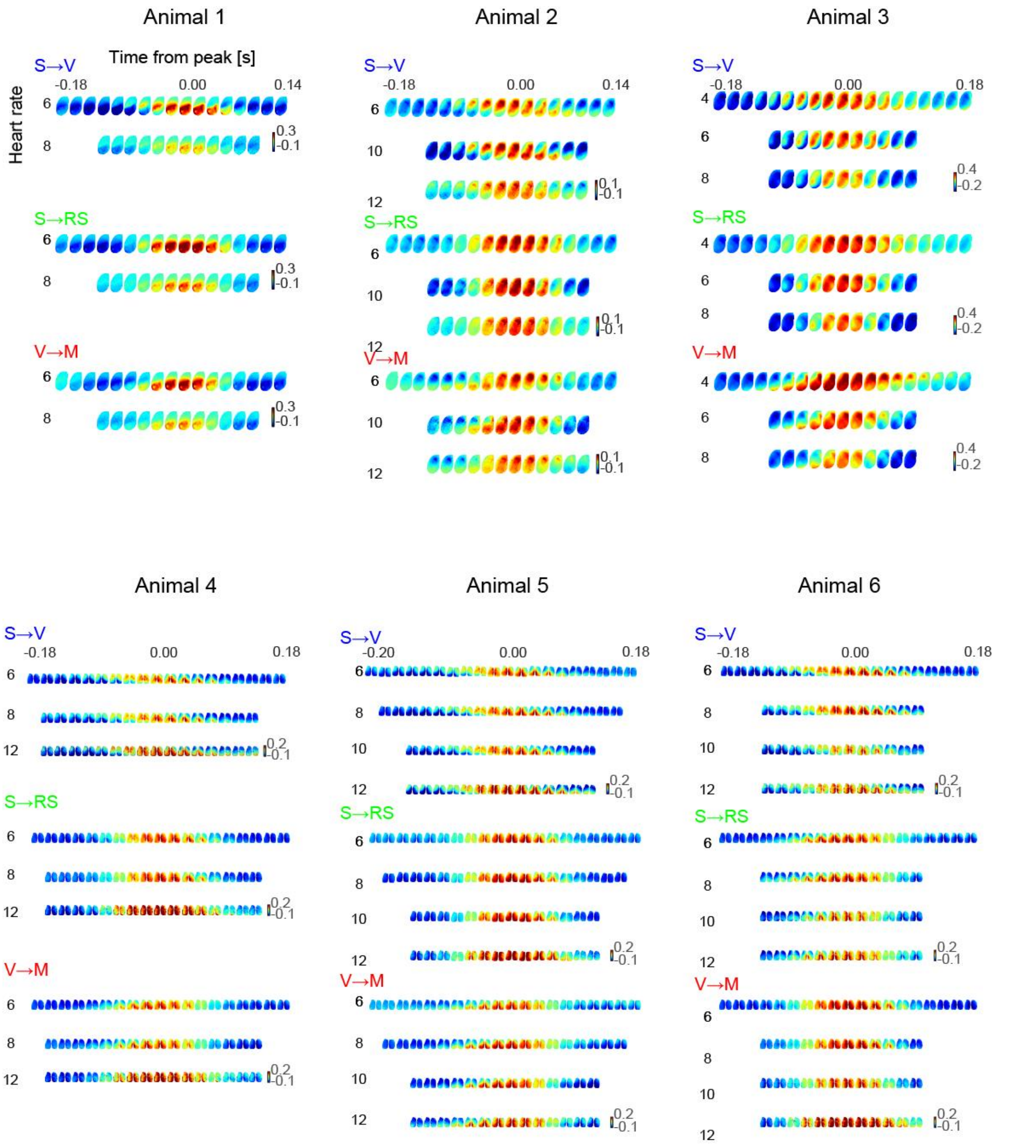

Figure S4, related to Figure 4. Expansion of Figure 4A for all animals and motifs. Individual GWEs were aligned to their voltage peak time and averaged across events per motif.
